# Correction: Determination of the electronic structure of a dinuclear dysprosium single molecule magnet without symmetry idealization

**DOI:** 10.1039/c9sc90051a

**Published:** 2019-03-01

**Authors:** Mauro Perfetti, Maren Gysler, Yvonne Rechkemmer-Patalen, Peng Zhang, Hatice Taştan, Florian Fischer, Julia Netz, Wolfgang Frey, Lucas W. Zimmermann, Thomas Schleid, Michael Hakl, Milan Orlita, Liviu Ungur, Liviu Chibotaru, Theis Brock-Nannestad, Stergios Piligkos, Joris van Slageren

**Affiliations:** a Institut für Physikalische Chemie , Universität Stuttgart , Pfaffenwaldring 55 , D-70569 Stuttgart , Germany . Email: slageren@ipc.uni-stuttgart.de; b Institut für Organische Chemie , Universität Stuttgart , Pfaffenwaldring 55 , D-70569 Stuttgart , Germany; c Institut für Anorganische Chemie , Universität Stuttgart , Pfaffenwaldring 55 , D-70569 Stuttgart , Germany; d Laboratoire National des Champs Magnétiques Intenses (LNCMI-EMFL) , CNRS , UGA , 38042 Grenoble , France; e Institute of Physics , Charles University , Ke Karlovu 5 , 12116 Praja 2 , Czech Republic; f Theory of Nanomaterials Group , Katholieke Universiteit Leuven , Celestijnenlaan 220F , 3001 Leuven , Belgium; g Department of Chemistry , University of Copenhagen , Universitetsparken 5 , 2100 , Denmark

## Abstract

Correction for ‘Determination of the electronic structure of a dinuclear dysprosium single molecule magnet without symmetry idealization’ by Mauro Perfetti *et al.*, *Chem. Sci.*, 2019, **10**, 2101–2110.



## 

The Royal Society of Chemistry regrets that the footnotes ‡ and § are incorrect in the original article. The correct footnotes are presented below.

‡ These authors contributed equally.

§ Current address: Department of Chemistry, University of Copenhagen, Universitetsparken 5, 2100, Denmark.

The Royal Society of Chemistry apologises for these errors and any consequent inconvenience to authors and readers.

